# Case Management Improves Anxiety And Depressive Symptoms For APS Clients at Case Closure

**DOI:** 10.1093/geroni/igaf122.224

**Published:** 2025-12-31

**Authors:** Courtney Reynolds, Samantha Tuft, Farida Ejaz, Jessica Bibbo, Traci Lee

**Affiliations:** Benjamin Rose Institute on Aging, Cleveland, Ohio, United States; Benjamin Rose Institute on Aging, Cleveland, Ohio, United States; Benjamin Rose Institute on Aging, Cleveland, Ohio, United States; Benjamin Rose Institute on Aging, Cleveland, Ohio, United States; Utah Department of Health & Human Services, Salt Lake City, Utah, United States

## Abstract

Caretaker neglect is the second most common type of abuse in the United States. In Utah, 74% of cases of caretaker neglect investigated by Adult Protective Services (APS) result in an ‘inconclusive disposition.’ With federal funding, practitioners from Utah APS collaborated with researchers from Benjamin Rose to develop and evaluate a telephone-based case management intervention for APS clients and their caretakers when there was an inconclusive finding of caretaker neglect. The goal was to prevent future reports of caretaker neglect or abuse. Using a four-month, pre-post design, care coordinators interviewed study participants, developed individualized care plans and referred to services and supports, making modifications as needed. In total, 95 clients and 83 alleged perpetrators consented to participate in the study. This presentation focuses on 54 clients who completed the baseline and posttest interviews. The average age of APS clients was 61 years (range: 18– 89), 56% were male, 26% lived in a rural/frontier area, and 24% participated with their alleged perpetrator. APS clients experienced a significant pre-post reduction in depressive symptoms using the Patient Health Questionnaire-2 (p = .007) and anxiety symptoms using the Generalized Anxiety Disorder-2 (p = .049). Discussion will focus on the impact of providing case management on APS clients’ improved anxiety and depressive symptoms at case closure. Implications for policy, research and practice include the value of multidisciplinary collaborations to develop interventions to prevent future caretaker neglect, and possibly other forms of abuse, neglect and exploitation.

